# Double Trouble: Acquired QTc Prolongation in a Patient on Denosumab and Amiodarone

**DOI:** 10.7759/cureus.91229

**Published:** 2025-08-29

**Authors:** Aamir Shaikh, Caleb W Brown, Sanskrita Nemalikanti, Zamir Shaikh, Venkata Pulivarthi

**Affiliations:** 1 Department of Internal Medicine, East Tennessee State University Quillen College of Medicine, Johnson City, USA; 2 Department of General Surgery, East Tennessee State University Quillen College of Medicine, Johnson City, USA; 3 Department of Internal Medicine, Alabama College of Osteopathic Medicine, Huntsville, USA

**Keywords:** amiodarone, denosumab, drug-induced qt prolongation, electrolyte imbalance, hypocalcemia, long qt syndrome

## Abstract

Prolongation of the corrected QT (QTc) interval is a known risk factor for life-threatening ventricular arrhythmias and can result from medications, electrolyte imbalances, or both. We report the case of a 70-year-old female with atrial fibrillation on amiodarone and osteoporosis treated with denosumab who presented with dizziness, but without syncope, palpitations, or chest pain. She was hemodynamically stable with negative orthostatic vitals, and neurologic examination revealed a positive Romberg sign. Brain imaging was unremarkable. ECG demonstrated marked QTc prolongation >550 ms (reference range: <460 ms for women). Laboratory testing revealed hypocalcemia with total calcium of 8.3 mg/dL (reference: 8.5-10.5 mg/dL) and ionized calcium of 1.05 mg/dL (reference: 4.5-5.3 mg/dL). Given her recent denosumab infusion within the prior week and ongoing amiodarone use, acquired long QT syndrome secondary to drug-induced hypocalcemia was suspected.

The patient received intravenous calcium gluconate, with resolution of symptoms and normalization of calcium levels. Amiodarone was discontinued and replaced with metoprolol. Her QTc improved to 486 ms on repeat ECG. The patient was advised to discontinue denosumab and follow up for alternative osteoporosis management. This report illustrates a rare but clinically significant interaction between denosumab-induced hypocalcemia and amiodarone, resulting in acquired long QT syndrome. It highlights the importance of monitoring electrolytes and cardiac conduction in patients receiving QT-prolonging and calcium-altering therapies. Early recognition and intervention are essential to prevent potentially fatal arrhythmic complications.

## Introduction

Denosumab is a monoclonal antibody that inhibits receptor activator of nuclear factor kappa-Β ligand (RANKL), thereby suppressing osteoclastic bone resorption. While effective in treating osteoporosis, this mechanism disrupts calcium mobilization from bone into the bloodstream, potentially leading to hypocalcemia [1,2]. The risk is particularly pronounced in individuals with high bone turnover or vitamin D deficiency, as the inhibition of osteoclast activity increases reliance on intrinsic calcium stores, and osteoblastic sequestration further lowers serum calcium levels [2,3]. Although compensatory elevations in parathyroid hormone (PTH) may occur, they are often insufficient to counteract denosumab’s pharmacologic effects [2,4]. The risk of hypocalcemia is further elevated in vulnerable populations, including those with chronic kidney disease (CKD), where disruptions in mineral metabolism compound the effects of denosumab [5,6].

Hypocalcemia can prolong the QT interval on ECG by interfering with the cardiac action potential, particularly the plateau phase (phase 2), which is dependent on calcium influx [7,8]. The reduction in extracellular calcium impedes inward calcium current during depolarization and delays repolarization, resulting in prolonged phases 2 and 3. This electrical instability predisposes to early afterdepolarizations and increases the risk for potentially fatal arrhythmias such as torsades de pointes [8,9].

Concomitant use of QT-prolonging medications, such as amiodarone, can amplify this risk. Amiodarone prolongs cardiac repolarization by blocking the rapid delayed rectifier potassium current (IKr), directly increasing the QTc interval [10,11]. When paired with hypocalcemia, the combination of impaired potassium efflux and diminished calcium influx poses a compounded threat to cardiac stability [8,12]. Elderly patients are particularly susceptible due to the prevalence of polypharmacy, underlying comorbidities, and age-related changes in renal and cardiac physiology [11,13]. We present a case of severe acquired QTc prolongation in a patient receiving both denosumab and amiodarone, highlighting a rare but clinically significant interaction with potentially life-threatening consequences.

This report was previously presented as a meeting abstract at the 2025 Dr. Jay W. Boland Research Day on April 16, 2025.

## Case presentation

**Figure 1:**
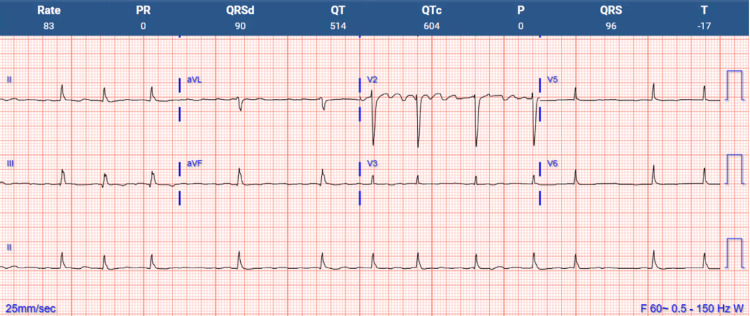
Initial ECG indicating severely prolonged QTc interval (>550 ms) ECG: electrocardiogram

A 70-year-old female with atrial fibrillation on amiodarone and osteoporosis on denosumab presented to the ED complaining of dizziness without syncope, palpitations, or chest pain. She was hemodynamically stable with negative orthostatic vitals. Neurologic examination revealed a positive Romberg without other cerebellar deficits. MRI showed an incidental vascular loop within the internal auditory canal without acute ischemic changes. Benign paroxysmal positional vertigo, Meniere’s disease, vestibular neuritis, and giant cell arteritis were ruled out based on history and physical examination. Laboratory tests revealed hypocalcemia (8.3 mg/dL; reference range: 8.5-10.5 mg/dL) with reduced ionized calcium (1.05 mg/dL; reference range: 4.5-5.3 mg/dL). ECG showed a QTc interval >550 ms (reference range: <450 ms for men, <460 ms for women), suggesting acquired long QT syndrome due to hypocalcemia (Figure 1).

**Figure 2:**
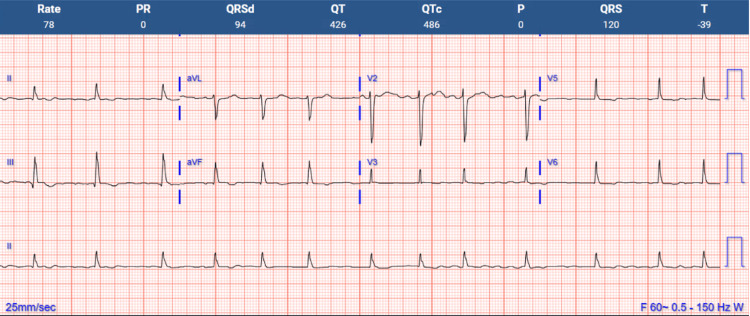
Post-amiodarone cessation ECG showing significantly improved QTc interval ECG: electrocardiogram

Given the patient's recent denosumab infusion (administered less than a week before presentation) and ongoing amiodarone use, drug-induced QTc prolongation was suspected. She was treated with IV calcium gluconate, leading to symptomatic improvement and calcium normalization. Amiodarone was replaced with metoprolol. Following these interventions, QTc improved to 486 ms (Figure 2). The patient was advised to transition from denosumab to an alternative osteoporosis therapy during outpatient follow-up.

## Discussion

Patients receiving medications such as denosumab and amiodarone require close monitoring of electrolytes and cardiac conduction due to the individual and combined risks of QTc prolongation. Denosumab can induce hypocalcemia, particularly in those with high bone turnover or impaired calcium homeostasis, while amiodarone, through its class III antiarrhythmic properties, prolongs cardiac repolarization [14]. When both mechanisms act concurrently, as in this case, the risk of cardiac arrhythmia may be significantly amplified. Importantly, accurate diagnosis relies on a thorough biochemical assessment. Measuring ionized calcium, the physiologically active form, is crucial in evaluating calcium status, particularly in patients with altered albumin levels or protein-binding abnormalities [15]. Reliance on total calcium alone may mask significant hypocalcemia and delay targeted intervention, thereby increasing the risk of arrhythmic complications such as torsades de pointes.

Management of hypocalcemia should be tailored to clinical severity and electrocardiographic findings. In symptomatic patients or those with marked QTc prolongation, IV calcium replacement is the preferred acute therapy. Calcium gluconate is favored over calcium chloride due to its lower risk of tissue necrosis when administered peripherally. Initial dosing typically involves 1-2 grams infused over 10-20 minutes, with the option for continuous infusion if calcium levels remain low. Care must be taken to avoid co-infusion with phosphate or alkaline solutions, which may cause precipitation [16]. Equally important is identifying and discontinuing offending agents. In this case, denosumab was recognized as the likely trigger for hypocalcemia, while amiodarone exacerbated QTc prolongation. The patient’s improvement following IV calcium and withdrawal of amiodarone underscores the effectiveness of both targeted electrolyte correction and judicious pharmacologic review in managing acquired long QT syndrome.

This case also highlights broader clinical implications for polypharmacy management. Denosumab is associated with hypocalcemia in 3-10% of patients, with severe or fatal outcomes reported in high-risk populations such as those with renal dysfunction. Guidelines recommend baseline and serial calcium monitoring, especially in patients with predisposing conditions [17]. Additionally, denosumab should not be co-administered with other hypocalcemia-inducing agents like etelcalcetide, given the risk of severe serum calcium depletion. Amiodarone, while effective for atrial fibrillation, is a high-risk agent for QTc prolongation, particularly when used with other medications that impair repolarization. Combinations with drugs like digoxin, macrolides, fluoroquinolones, and other antiarrhythmics have been associated with adverse outcomes, including torsades de pointes. Routine QTc monitoring is essential, and amiodarone is generally contraindicated in patients with pre-existing QTc prolongation or congenital long QT syndromes [18]. The combination of denosumab and amiodarone in this patient created a pharmacologic substrate conducive to both electrolyte derangement and electrical instability, exemplifying the risks of polypharmacy without coordinated monitoring.

To our knowledge, this is the first reported case of acquired QTc prolongation resulting from the combination of denosumab and amiodarone. While both agents independently carry risks of QTc prolongation - denosumab via hypocalcemia and amiodarone via potassium channel blockade - there are no prior reports documenting this specific drug interaction. However, several cases in the literature have demonstrated that amiodarone, when combined with other QTc-prolonging agents, can lead to life-threatening arrhythmias [19]. These include documented instances of torsades de pointes or cardiac arrest when amiodarone was used in combination with agents such as fluoxetine, metronidazole, ciprofloxacin, sotalol, clarithromycin, and fluconazole, often involving CYP3A4-related interactions and additive QTc effects.

These reports collectively underscore the well-established risk of additive QTc prolongation in the setting of polypharmacy involving amiodarone. Our report builds upon this body of literature by identifying denosumab-induced hypocalcemia as a novel co-contributor to this risk, reinforcing the need for clinical vigilance when combining QTc-prolonging or electrolyte-altering therapies.

## Conclusions

This report underscores several key considerations for clinicians managing patients on QT-prolonging and calcium-altering therapies. First, denosumab-induced hypocalcemia, while uncommon, can significantly disrupt cardiac repolarization and lead to acquired long QT syndrome, particularly when paired with other QT-prolonging agents such as amiodarone. Second, this report illustrates the additive risk posed by polypharmacy in elderly patients with multiple comorbidities, emphasizing the importance of routine electrolyte and ECG monitoring in high-risk populations. Finally, the patient’s rapid clinical and electrocardiographic improvement following targeted calcium replacement and medication adjustment highlights the reversibility of this condition with timely recognition and intervention. This report reinforces the need for proactive risk assessment and coordinated medication management to prevent potentially life-threatening arrhythmias.
